# Life or Death: Prognostic Value of a Resting EEG with Regards to Survival in Patients in Vegetative and Minimally Conscious States

**DOI:** 10.1371/journal.pone.0025967

**Published:** 2011-10-05

**Authors:** Alexander A. Fingelkurts, Andrew A. Fingelkurts, Sergio Bagnato, Cristina Boccagni, Giuseppe Galardi

**Affiliations:** 1 BM-Science – Brain and Mind Technologies Research Centre, Espoo, Finland; 2 Neurorehabilitation Unit, Rehabilitation Department, Fondazione Istituto “San Raffaele - G. Giglio,” Cefalù, Palermo, Italy; 3 Neurophysiology Unit, Rehabilitation Department, Fondazione Istituto “San Raffaele - G. Giglio,” Cefalù, Palermo, Italy; Cuban Neuroscience Center, Cuba

## Abstract

**Objective:**

To investigate the potentially prognostic value of a resting state electroencephalogram (EEG) with regards to the clinical outcome from vegetative and minimally conscious states (VS and MCS) in terms of survival six months after a brain injury.

**Methods:**

We quantified a dynamic repertoire of EEG oscillations in resting condition with eyes closed in patients in VS and MCS. The exact composition of EEG oscillations was assessed by analysing the probability-classification of short-term EEG spectral patterns.

**Results:**

Results demonstrated that (a) the diversity and the variability of EEG for Non-Survivors were significantly lower than for Survivors; and (b) a higher probability of mostly delta and slow-theta oscillations occurring either alone or in combination were found during the first assessment for patients with a bad outcome (i.e., those who died) within six months of an injury compared to patients who survived. At the same time, patients with a good outcome (i.e., those who survived) after six months post-injury had a higher probability of mostly fast-theta and alpha oscillations occurring either alone or in combination during the first assessment when compared to patients who died within six months of an injury.

**Conclusions:**

Resting state EEGs properly analysed may have a potentially prognostic value with regards to the outcome from VS or MCS in terms of survival six months after a brain injury.

**Significance:**

This work may have implications for clinical care, rehabilitative programmes and medical–legal decisions for patients with impaired consciousness states after being in a coma due to acute brain injuries.

## Introduction

Severe brain injury presents serious social and economic problems [1], [2] that warrant further research. Thanks to advances in critical care, patients increasingly survive severe brain injury, resulting in a greater incidence and prevalence of patients in vegetative and minimally conscious states (VS and MCS respectively) [3]. A vegetative state (VS) is “a clinical condition of unawareness of self and environment in which the patient breathes spontaneously, has a stable circulation, and shows cycles of eye closure and opening which may simulate sleep and waking” [4]. A minimally conscious state (MCS) is “a condition of severely altered consciousness in which minimal but definite behavioural evidence of self or environmental awareness is demonstrated. In MCS, cognitively mediated behaviour occurs inconsistently, but is reproducible or sustained long enough to be differentiated from reflexive behaviour” [5].

Prognostic accuracy for patients in VS and MCS poses a serious ethical concern because treatment decisions typically include the possibility of life-support being withdrawn [6], [7]. Currently, prognosis of the outcome is determined primarily through diagnosis (VS or MCS) and also by aetiology of brain injury (traumatic, vascular or anoxic) and the age of the patient. However, misdiagnoses of VS and MCS are common and have been shown to be as high as 37–43% [8]–[11]. Therefore, additional objective measurement tools are needed to achieve better prognostic accuracy for patients in VS and MCS.

It has been shown previously (a) that electroencephalogram (EEG) may be useful in the evaluation of the level of consciousness recovery [12]–[14] and (b) that the background frequencies of the EEG measured with power spectral analysis are highly correlated with the Glasgow Coma Scale and this correlation improves with increasing time post-injury [15]. These studies suggest that EEG may have a prognostic value in predicting the outcome of a traumatic coma. Indeed, it has been demonstrated that a pattern of unvarying activity with the major frequency component in the delta–slow-theta (1–3 Hz) band predicts poor prognosis [16]–[18], whereas a peak in the alpha or fast-theta frequency band indicates a good outcome from traumatic coma [19], [20]; see also [13].

However, there is a lack of EEG studies which attempt to evaluate prognosis of the outcome from VS or MCS in terms of survival probability over time. To our knowledge there is only one study which suggested association between EEG patterns and death in VS patients [21]. It therefore remains an open question whether certain EEG features can predict the future survival of patients with disorders of consciousness regardless of whether they are in VS or MCS. Before the prognostic markers that allow clinicians to predict the chances of future survival in the *individual* patient in VS or MCS can be validated, these markers must be identified at the *population* level.

Therefore, the **aim** of the present study was twofold: (a) to explore the potential prognostic value of resting EEG with regards to clinical outcome from VS or MCS in terms of survival six months after a brain injury and (b) to identify potential candidates for prognostic EEG markers that allow researchers to predict the chances of future survival in VS or MCS, at least at group level.

## Methods

### Ethics Statement

Patients’ relatives and/or legal guardians gave their written informed consent; the study and the consent procedure were performed according to the Declaration of Helsinki and approved by the Ethical Committee of the Fondazione Istituto San Raffaele G. Giglio (Cefalù, Italy).

### Subjects

The study was performed on 21 non- or minimally communicative patients with severe brain injuries suffering from different consciousness disorders (Table 1), admitted to the Neurorehabilitation Unit of Fondazione Istituto “San Raffaele - G. Giglio” to carry out an intensive neurorehabilitation program.

**Table 1:** Basic demographic and clinical characteristics of the patients.

Patient ID	Age	Gender	Type of consciousness disorder	Aetiology	TC/MRI findings (in the acute phase)	Time (in days) between acute event and EEG recording	Drugs	LCF at the EEG recording day	Outcome after 6 months
**SURVIVORS (survived after 6 months)**									
1	38	M	MCS	Trauma	Subdural and epidural hematoma in the right hemisphere; right fronto-temporal intraparenchymal hemorrhage; right fronto-temporal cortical contusions	36	None	3	S
2	36	M	VS	Trauma	Left parieto-temporal intraparenchymal hemorrhage; several intraparenchymal micro-hemorrhages	36	PB 100	2	S
3	35	M	VS	Trauma	Diffuse axonal injury; right temporal cortical contusion	42	None	1	S
4	28	M	VS	Trauma	Subdural and epidural hematoma in the right hemisphere	46	None	2	S
5	19	F	MCS	Trauma	Subdural hematoma in the right hemisphere; bilateral frontal cortical contusions	74	None	3	S
6	55	M	VS	Trauma	Cortical contusions in the frontal lobes and in the right temporal lobe; subdural hematoma; diffuse axonal injury	37	None	2	S
8	19	M	VS	Trauma	Intraparenchymal microhemorrhages in the right frontal, temporal and parietal lobes; diffuse axonal injury	14	None	2	S
9	64	F	MCS	Trauma	Cortical contusions in the temporal lobes and in the right parietal lobe	56	VPA 1500	3	S
10	35	M	VS	Vascular	Left subarachnoid hemorrhage and left temporo-parieto-occipital ischemia (due to vasospasm)	32	None	2	S
11	61	F	MCS	Vascular	Intraparenchymal hemorrhage in the right parietal lobe	77	None	3	S
13	29	F	MCS	Vascular	Fronto-temporo-parietal intraparenchymal hemorrhage in the right hemisphere	45	VPA 600	3	S
15	79	F	VS	Vascular	Intraparenchymal hemorrhage in left parieto-occipital region	77	LTG 200, PB 100	2	S
18	66	M	VS	Vascular	Right fronto-temporo-parietal intraparenchymal and subarachnoid hemorrhage	72	PB 100	1	S
19	57	M	VS	Vascular	Brainstem hemorrhage	87	PB 100	2	S
20	16	M	VS	Anoxia		92	None	2	S
Mean±st.d Summary	42.5±19.7		MCS: 33%, VS: 67%	T: 53%, NT: 47%		54.9±23	N: 60%, D: 40%	2.2±0.7	
**NON-SURVIVORS (died after 6 months)**									
7	14	M	VS	Trauma	Subdural hematoma in the left hemisphere; widespread intraparenchymal microhemorrhages	89	PB 100	2	D (sepsis)
12	41	M	VS	Vascular	Fronto-temporo-parietal intraparenchymal hemorrhage in the left hemisphere	44	None	1	D (sepsis)
14	60	F	MCS	Vascular	Subdural hematoma in the left hemisphere	67	CBZ 800, PB 100	3	D (sepsis)
16	70	M	MCS	Vascular	Left temporo-parietal ischemia	44	None	3	D (myocardial infarction)
17	50	M	VS	Vascular	Hemorrhage in the right putamen	79	None	2	D (sepsis)
21	68	M	VS	Anoxia		63	None	1	D (respiratory failure)
Mean±st.d Summary	50.5±21		MCS: 33%, VS: 67%	T: 17%, NT: 83%		64±18	N: 67%, D: 33%	2±1.0	

M - male, F - female, MCS - minimally conscious state, VS - vegetative state, T - traumatic aetiology, T - non-traumatic aetiology, N - no drug, D - drugs, LCF - level of cognitive functioning scale, VPA - valproic acid, CBZ - carbamazepine, PB - phenobarbital, LTG - lamotrigine, S - survived, D - died.

On admission all patients underwent a thorough and comprehensive clinical neurological examination. The diagnosis of VS and MCS was made according to currently accepted diagnostic criteria [22]–[24]. Additionally, the Levels of Cognitive Functioning (LCF) score [25] was assessed on the day of admission and three days later when the EEG was recorded. We chose to use the LCF scale instead of the Glasgow Outcome Scale (GOS) [26], the Glasgow Coma Scale [27] or the JFK Coma Recovery Scale [28] because LCF evaluates not only behavioural patterns but also cognitive functions and LCF has been found better related to the presence of EEG abnormalities [14]. The LCF scale has different grades ranging from 1 to 8 (1 – patient does not respond to external stimuli and/or command; 8 – patient is self-oriented and responds to the environment but abstract reasoning abilities decrease relative to pre-morbid levels).

Based on the LCF score 14 of the patients were classified as being in a vegetative state (VS) (LCF: 1–2) and the remaining 7 patients were classified as being in a minimally conscious state (MCS) (LCF: 3). In order to reduce the variability of clinical evaluation, LCF scores were assigned to all patients only if they were unchanged for the day of admission and the day of the EEG registration; otherwise, patients were excluded from the study. Other criteria for excluding patients were (a) any acute comorbidity or unstable vital signs; (b) obvious communicating or obstructive hydrocephalus; (c) a history of neurological disease before admission; and (d) severe spasticity (causing constant EMG artefacts). Inclusion criteria for the patients included (a) confirmation of diagnosis of VS or MCS according to the clinical definitions [5], [22], [23]; (b) LCF  =  1–2 for VS and 3–4 for MCS patients; (c) less than 3 months after the acute brain event onset; and (d) first-ever acute brain event. None of the chosen patients were excluded because the scores for all the patients in the study remained unchanged from the day of admission to the day that the EEG was recorded.

For the purpose of this study, all patients, both in a VS and MCS, were categorised according to the outcome (survived or died) which was assessed 6 months after the brain injury (Table 1). *Survivors group* (*n*  =  15) contained 5 (33%) patients in MCS and 10 (67%) patients in VS; *Non-Survivors group* (*n*  =  6) contained 2 (33%) patients in MCS and 4 (67%) patients in VS.

### EEG recording

Spontaneous electrical brain activity was recorded with a 21-channel EEG data acquisition system (Neuropack electroencephalograph; Nihon Kohden, Tokyo, Japan). EEG data was collected (cephalic reference – mean of the signals from C3 and C4 electrodes; 0.5–70 Hz bandpass; 200 Hz sampling rate; around 30 min) in patients during a waking resting state (eyes-closed) from 19 electrodes positioned in accordance with the International 10–20 system (i.e. O_1_, O_2_, P_3_, P_4_, P_z_, T_5_, T_6_, C_3_, C_4_, C_z_, T_3_, T_4_, F_3_, F_4_, F_z_, F_7_, F_8_, Fp_1_, Fp_2_). The impedance of recording electrodes was monitored for each subject and was always below 5 kΩ. To monitor eye movements, vertical and horizontal electrooculograms (0.5–70 Hz bandpass) were also recorded.

The EEG recordings for all patients were performed during the late morning within three days after admission. EEG recordings were started in all cases only if patients spontaneously had their eyes open, the eyelids were then closed by hand. The eyes were closed by hand all the time until the end of EEG registration. At the end of the recordings all patients opened their eyes spontaneously thus suggesting that the level of vigilance (i.e. capability to open eyes) was unchanged compared to the onset of the EEG. In order to keep a constant level of vigilance, an experimenter monitored patients’ EEG traces in real time, looking for signs of drowsiness and the onset of sleep (increase in “tonic” theta rhythms, K complexes and sleep spindles). The presence of an adequate EEG signal was determined by visual inspection of the raw signal on the computer screen. Even though it may be difficult to assess precisely the level of vigilance in patients in VS, preserved sleep patterns may be observed in the majority of patients in VS (for review see [29]).

### EEG-signal data pre-processing

The presence of an adequate EEG signal was determined by visually checking each raw signal. Epochs containing artefacts due to eye movements, significant muscle activity, and movements on EEG channels were marked and then automatically removed from any further analysis.

Artefact-free EEG signals were filtered in the 1–30 Hz frequency range. This frequency range was chosen because approximately 98% of spectral power lies within these limits [30]. Although it has recently been proposed that frequencies above 30 Hz (gamma band) may be functionally informative, there are a number of methodological issues which lead us to exclude frequencies above 30 Hz from the present analysis: (a) it was shown that volume conduction has little influence on the shape of the spectrum below around 25 Hz, however spatial filtering is significant for frequencies above 25 Hz [31]; (b) high-frequency spindles have a very low signal-to-noise ratio, which results in considerable noise contamination of the gamma band; (c) the dynamics of high-frequency effects may be a trivial by-product of power changes in lower frequencies [32]; (d) increased power in the gamma range may be due to the harmonics of activity in lower frequency ranges, and/or due to the ringing of filters by EEG spikes recurring at theta rates [33]; (e) the gamma band may be an artefact of (un)conscious micro-constrictions of muscles of the organism and/or face muscles [34]–[36]; (f) comprising just 2% of the spectral power [30], the contribution of high-frequency band to the spectrum cannot be significant; (g) Bullock et al. [37] demonstrated many “good” rhythms in the 2–25 Hz range which were mainly sinusoidal but did not find them in the 30–50 Hz band. In light of the above, there may be difficulties in carrying out a meaningful interpretation of effects at the high-frequency band regardless of how powerful or statistically significant they are.

DC drifts were removed using high pass filters (1 Hz cut-off).

For each patient a full EEG stream free from any artefacts was fragmented into consecutive one-minute epochs. All one-minute EEGs were split into two groups according to the outcome from VS or MCS (i.e. survived or died) which was assessed 6 months after the brain injury (Table 1): *Survivors group* (171 one-minute EEGs) and *Non-Survivors group* (53 one-minute EEGs). Within each group further data processing was performed for each separate one-minute portion of the signal. Due to the technical requirements of the tools used to process the data, EEGs were re-sampled to 128 Hz. This procedure should not have affected the results since 128 Hz sampling rate meets the Nyquist Criterion [38] of a sample rate greater than twice the maximum input frequency and is sufficient to avoid aliasing and preserve all the information about the input signal. This method was considered sufficient since the sampling rate of the source signals was significantly higher than required.

**Figure 1:**
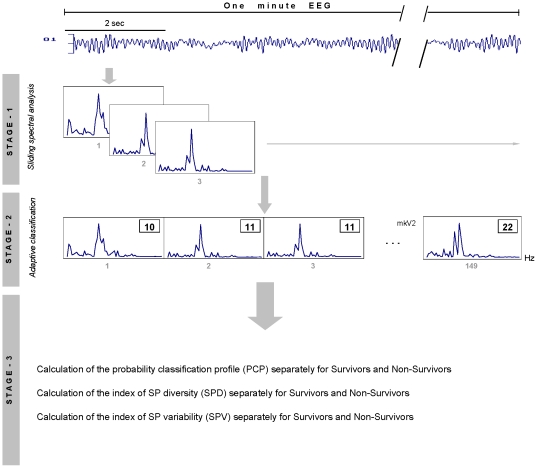
The scheme of data processing. *First stage*: A sliding spectral analysis was conducted separately for each patient and each one-minute EEG channel. O_1_  =  Left occipital EEG channel. *Second stage*: An adaptive classification of short-term spectral patterns (SP) was performed separately for each patient and each one-minute EEG channel. The small gray numbers under each SP represent the running numbers from 1 to 149 for a one-minute EEG. The number in the square represents the class to which a given SP was assigned during classification procedure. *Third stage*: (a) Probability-classification profile (PCP); (b) the Index of SP diversity (SPD) and (c) the Index of SP variability (SPV) were calculated separately for each patient and each one-minute EEG channel.

After re-sampling, EEG oscillations were identified. This procedure was undertaken in three stages (Fig. 1). During the first stage of EEG analysis, the data series from each EEG channel were separately divided into overlapping windows in order to capture EEG changing dynamics. EEG oscillations were quantified by calculation of individual short-term EEG spectral patters (SPs). Individual power spectra were calculated in the range of 1–30 Hz with 0.5-Hz resolution, using a Fast Fourier Transform with a 2-sec Hanning window shifted by 50 samples (0.39-sec) for each one-minute EEG channel (Fig. 1). According to previous studies, these values have proved to be the most effective for revealing oscillatory patterns from the signal [39], [40]. A sliding spectral analysis with overlapping segments, previously applied to EEG signals [41], [42], (a) takes the non-stationarity of the time series into account, (b) compensates for the effects of windowing and (c) prevents loss of information due to residual activity. Additionally, using overlapping intervals (which just means a different aggregation scheme) cannot add any artefactual information [43].

After calculation of EEG short-term SPs, the total number of individual SPs for each one-min EEG channel was 149 (Fig. 1).

During the second stage, with the help of a probability-classification analysis of the short-term EEG SPs (see [44] and Appendix in [45]), each SP was labelled according to the class index it belonged to. Sequential single EEG SPs were adaptively classified in each one-minute EEG channel using a set of standard SPs which were generated automatically from the EEG data itself. The selection was not arbitrary: a pool of SPs (*n*  =  685 102) was collated from all the SPs for all the EEG signals (all locations) for all patients. From this pool, all identical SPs with dominant power peaks (peaks that rise significantly above the general average) were counted automatically. The peak detection was based on normalising the SP to within-SP relative percentages of magnitude, where acceptance is achieved when the peak exceeds a given (60%) percent-magnitude (100% corresponds to the magnitude of the highest peak within the SP). According to the preliminary study, this value has proved to be the most effective for peak detection. The set of SPs with the highest count were the most probable candidates to form the “set of standard SPs.” Only those SPs with a minimum mutual correlation were selected. As a result, in this study the standard set included 32 SPs. Using a probability-classification procedure (see [44] and Appendix in [45]), each current SP was labelled according to the index of the class to which it belonged (Fig. 1). Hence, each one-minute EEG signal was reduced to a sequence of individually classified SPs.

During the third stage, probability-classification profiles (PCPs) of SPs for each channel of one-minute EEG in each patient were calculated. These PCPs were calculated by taking the relative number of cases of an SP type as a percentage of the total amount of all SPs within each EEG channel – presented as the histogram of relative presence of each SP type.

PCPs were averaged across all one-minute EEG signals and across 19 EEG channels separately for each patient within Survivors and Non-Survivors. It was expected that PCPs would make it possible to illustrate in general the composition of brain oscillations and their percent ratio (in SP description) for Survivors and Non-survivors.

In addition, two indices were calculated for each patient separately for each channel of each one-minute EEG:

1. An index of *SP diversity* (SPD) was estimated as a ratio of the number of SP types detected in a given one-minute EEG to the total number in the standard set (32 standard SPs  =  100%). SPD indicates how many different SP types participate in PCP.
2. An index of *SP variability* (SPV) is a percentage value that reflects how the set of distinct SP types changes across the three EEG sub-segments of twenty-seconds within a complete one-minute.

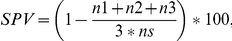
where *n*_i_, is the number of distinct SP types found in a 20-sec EEG segment *i*; *n*_s_ is the number of SP types found in all three 20-sec EEG segments. This index has a range from 0 (minimum variability) to 67 (maximum variability).

SPD and SPV were averaged across all one-minute EEG signals and across 19 EEG channels separately for each patient within Survivors and Non-Survivors.

### Statistics

The Wilcoxon *t*-test was used to reveal any statistically significant differences in the presence of each SP type in EEG between Survivors and Non-Survivors. Statistical significance was assumed where *p* <0.05. Since only the difference between pairs of conditions was of interest and we intended to assess each variable in its own right, no correction for multiple comparisons was necessary (for a detailed discussion, see [46], [47]). Differences in the demographic data were assessed either by Wilcoxon *t*-test or by Chi-square test.

## Results

### Demographical data

There were no statistically significant differences between Survivors and Non-Survivors groups in terms of age (*p*  =  0.33), time post brain injury (*p*  =  0.33), LCF score (*p*  =  0.67), as well as distribution of MCS and VS (33% of MCS and 67% of VS in both groups) and medicated vs. non-medicated patients (*p*  =  0.31) (Table 1).

### General description of EEG for Survivors and Non-Survivors

**Figure 2:**
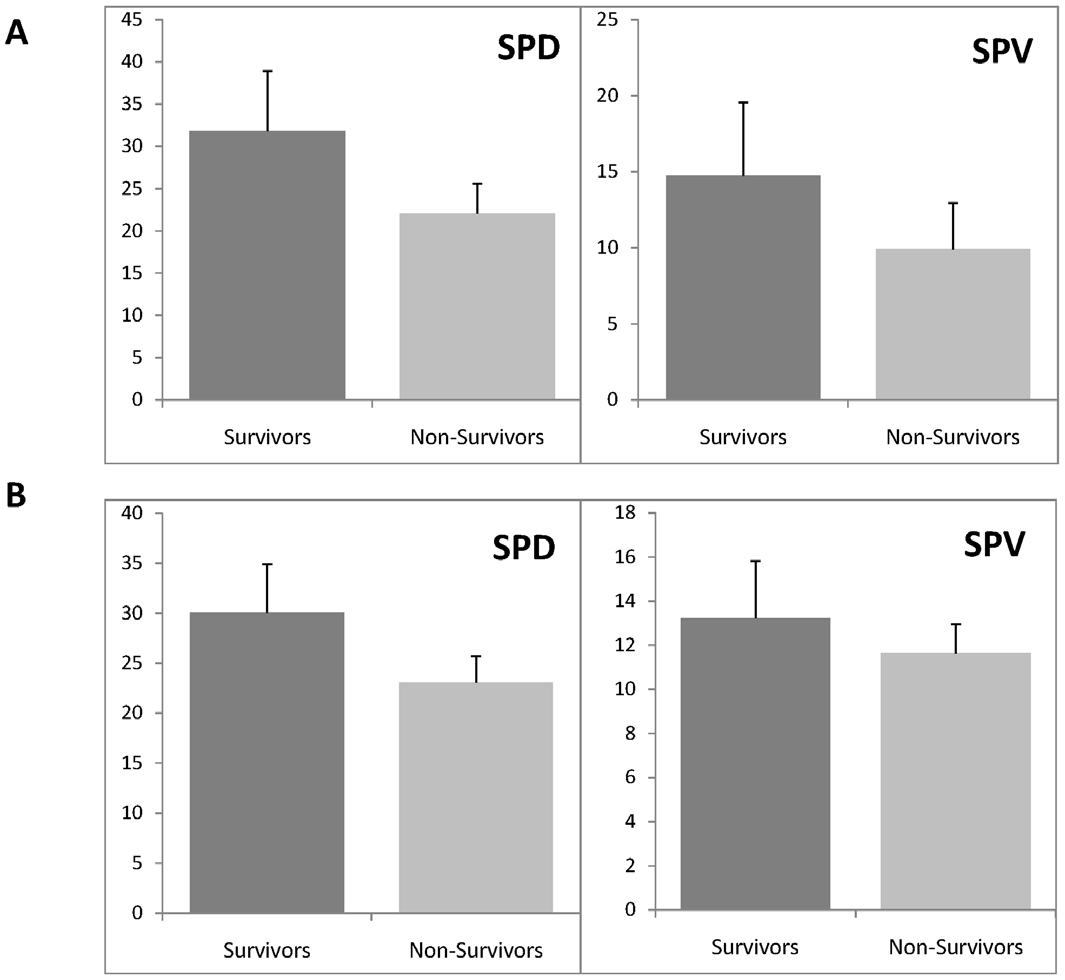
Index of SP diversity (SPD) and Index of SP variability (SPV) for Survivors and Non-Survivors. (A) Complete groups (medicated + unmedicated) of patients. Data averaged across 171 (for Survivors) and 53 (for Non-Survivors) one-minute EEG signals and across 19 EEG channels and presented as mean ± standard deviation. (B) Sub-groups of unmedicated patients only. Data averaged across 124 (for Survivors) and 33 (for Non-Survivors) one-minute EEG signals and across 19 EEG channels and presented as mean ± standard deviation. SP – spectral pattern; Survivors – patients who survived 6 months after brain injury; Non-Survivors – patients who died 6 months after brain injury.

Both the diversity and variability of SP types (indexed by SPD and SPV respectively, see Methods) were significantly higher for the Survivors than for the Non-Survivors (*p* <0.03 −*p* <0.001, Fig. 2.A).

To check whether the effects of different sedative drugs on the EEGs could affect these differences between the Survivors and Non-Survivors groups, we repeated an analysis for only medication-free patients. Analysis of medication-free patients demonstrated the same differences between Survivors and Non-Survivors groups (*p* <0.03, Fig. 2.B) as for the complete groups (medicated + non-medicated patients).

### Composition of multiple EEG oscillations for Survivors and Non-Survivors

To estimate which EEG oscillations within a broad frequency range (1–30 Hz) occurred more or less frequently for Survivors and Non-Survivors, we examined the probability of the occurrence of SP types (which characterise EEG oscillations or their mixture).

**Figure 3:**
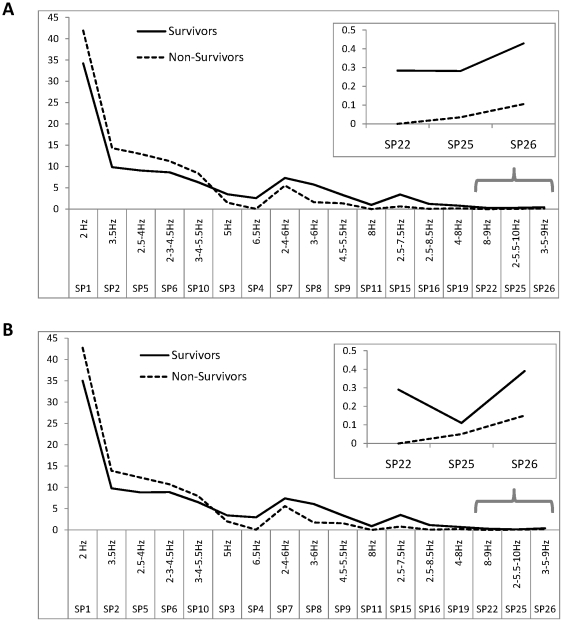
Probability-classification profiles (PCPs) for **Survivors and Non-Survivors.** The *X*-axis displays the labels of spectral pattern (SP) types and their main frequency peaks (Hz). The *Y*-axis displays the share of the corresponding SPs in the percentage from the total number of the classified SPs. A line graphic was chosen instead of a bar for ease of comparison. (Note that *X*-axis consists of discrete values, all the in-between values are meaningless). (A) Complete groups (medicated + unmedicated) of patients. Data averaged across 171 (for Survivors) and 53 (for Non-Survivors) one-minute EEG signals and across 19 EEG channels. (B) Sub-groups of only unmedicated patients. Data averaged across 124 (for Survivors) and 33 (for Non-Survivors) one-minute EEG signals and across 19 EEG channels. Survivors – patients who survived 6 months after brain injury; Non-Survivors – patients who died 6 months after brain injury.

Comparative analysis of the PCPs demonstrated that Survivors and Non-Survivors differed from each other according to the estimated probability of the occurrence of SP types in PCPs. We have found that lower probability values of the occurrence of mostly delta, theta_1_ and theta_2_ oscillations either alone or in combination (*p* <0.03−*p* <0.001 for different SP types) and higher probability values of the occurrence of mostly theta_3_, alpha_1_ and alpha_2_ oscillations (*p* <0.05 − *p* <0.00003 for different SP types) were present in patients who survived after six months post-injury as compared to patients who died (Fig. 3.A).

To check whether the effects of different sedative drugs on the EEGs could affect these differences between Survivors and Non-Survivors groups, we repeated the analysis for only medication-free patients. Analysis for medication-free patients demonstrated the same differences between Survivors and Non-Survivors groups (*p* <0.04 − *p* <0.002 for different SP types, Fig. 3.B) as for complete groups (medicated + non-medicated patients).

## Discussion

### Demographic factors

Since there were no significant differences between the Survivors and Non-Survivors groups in terms of age, time post brain injury, LCF score, distribution of MCS and VS and distribution of medicated vs non-medicated patients, all these factors could not be responsible for the differences in EEG parameters found between the Survivors and Non-Survivors groups.

Even though the percentage of patients with pharmacological therapies did not significantly differ between the groups, it could be argued that the effects of different sedative drugs on the EEGs could give rise to the differences between Survivors and Non-Survivors groups. To verify this supposition, we repeated the analysis for medication-free patients only. This analysis of medication-free patients demonstrated the same differences between Survivors and Non-Survivors groups (Fig. 2.B, Fig. 3.B) as for the complete groups (medicated + non-medicated patients), thus rejecting the assumption that our results could be affected by sedative drugs.

Patients with epidural or subdural hematomas may potentially affect EEG results due to the lower conductivities from brain to scalp. However, the time between brain injury and EEG recording was >1 month for patients with epidural or subdural hematomas. We assume that sufficient time had lapsed for the hematoma to be reabsorbed.

### General description of EEG for Survivors and Non-Survivors

This study demonstrated that the diversity and variability (indexed by SPD and SPV respectively, see Methods) of EEG were significantly lower for Non-Survivors than for Survivors. Both these indices represent different but converging aspects of the temporal dynamics of variability in SP types, reflecting the dynamic repertoire of EEG oscillatory microstates [48].

Based on the functional significance of individual SPs [48], [49], the type of SP may represent a particular oscillatory state of neurodynamics in a given cortical area. If this is the case, then the diversity of SP types represents the range of the probable states. Thus, the results obtained suggest that cortical areas for Non-Survivors were characterised by a considerably reduced dynamic repertoire of the probable states (decrease in the number of SP types and their temporal variability in EEGs). This represents the increased rigidity in the brain activity for Non-Survivors.

Therefore, both findings may suggest that EEG for Non-Survivors is more ordered and thus is expected to have lower entropy than EEG for Survivors. This supposition is supported by the work of Sarà and colleagues [21], where the authors demonstrated that death of VS patients was associated with the lowest approximate entropy (ApEn) values (see also [50]). Taken together, this data pointed to a conclusion that the brain’s operations in Non-Survivors were completed less dynamically than in Survivors and there is a transition to a less differential organisation of spectral relations, where neural elements become less independent. The oscillatory activity of neuronal pools, reflected in characteristic EEG rhythms, constitutes a mechanism by which the brain can regulate state changes in selected neuronal networks that lead to a qualitative transition between modes of information processing [51]. Therefore, a reduced dynamic repertoire of EEG oscillatory states in Non-Survivors signifies a decrease in the number of information processing modes, thus suggesting a reduction of brain information processing.

How these deficits in cortical information processing can explain mortality? To answer this question more studies are needed. However some speculative explanation can be offered.

Several studies demonstrated that the coordination and communication in and between the autonomic vegetative systems and the brain occur with tuned frequencies in the range of EEG oscillations suggesting the existence of oscillatory links in the brain and all organs of the body (for the review and discussion see [52], see also [53]). Basar [52] suggested that such mutual resonances form a coordinated dynamic system which maintains survival functions, such as normative values of blood pressure, respiratory rhythms, cardiac pacemakers and body temperature (see also [54], [55]). Additionally, the link between several factors of the immune system and EEG activity was reported [56]–[60]. Since dynamics of physiologic variables (autonomic and immune systems) depends on the dynamics of brain activity, it is reasonable to hypothesize that reduced variability in neural networks should cause a decrease in variability of such physiologic variables as autonomic and immune functions. Indeed it was demonstrated that a widespread brain injury causing a derangement in neural networks leads in VS patients to a reduced complexity (measured by ApEn) of EEG [61] and of heart rate [62] when compared with healthy subjects. It seems that decreased EEG variability is paralleled with decrease in variability of other physiologic variables (autonomic and immune systems) which result in reduced physiological adaptability and immunity. Reduced physiological adaptability and immunity in its turn may contribute to mortality.

In summary, both findings reflecting a considerable decrease in EEG variability for Non-Survivors corroborate the well-known fact that a loss of variability in the majority of physiological measures is associated with increased mortality in patients [63]–[65]. However, as far as we are aware, this study is the first to demonstrate decreased EEG variability (in terms of the number and temporal variability of SP types) for Non-Survivors compared to Survivors regardless of whether they are in VS or MCS.

### Composition of multiple EEG oscillations for Survivors and Non-Survivors

The results of this study show that higher probability values of the occurrence of mostly delta and slow-theta oscillations alone or their combinations were found during the first assessment for patients who showed a bad outcome (i.e. died) six months after the brain injury compared to patients who survived. At the same time, patients who showed a good outcome (i.e. survived) six months after the brain injury demonstrated higher probability values of the occurrence of mostly fast-theta and alpha oscillations alone or their combinations during the first assessment when compared to patients who died six months after the brain injury.

Even in cases where the same EEG oscillations were present in EEGs in both groups, oscillations in Survivors’ EEGs were characterised by faster frequencies than for Non-Survivors’ EEGs. This could reflect a higher activation level in neurons and in the cortical neuronal networks of Survivors when compared to Non-Survivors.

The results observed are in line with the previous data which suggested that increasing power in the alpha and theta bands may predict survival [66]. At the same time, the findings of this study substantially extend the results of conventional spectral analysis: our analysis revealed changes in the total amount of time (percentage of EEG segments) that a particular type of brain oscillations was “on”, rather than the changes in its amplitude or power. Our results are further supported by other studies which reported that an increase of slow activity and decrease of alpha activity in EEG is associated with an increased risk of mortality [67]–[69].

It is well known that an increase in the amount of delta and slow-theta activity in resting awake EEG is usually proportional to the degree of pathological processes and reflects encephalopathy and/or structural lesions [70], whereas alpha activity reflects a better functional state of the brain [71]. Perhaps such differences in EEG oscillatory descriptors of brain functional states found during the first assessment contribute to the outcome observed after six months. Our hypothesis is in line with previous studies which demonstrated that a pattern of unvarying activity with the major frequency component in the delta–slow-theta (1–3 Hz) band predicts poor prognosis [16], [18], whereas a peak in the alpha or fast-theta frequency band indicates a good outcome from a traumatic coma [19], [20], [72]. Hence our results suggest that the prognostic value of these EEG oscillations also remains after a coma when patients are in MCS or VS.

In summary, the observed results suggest that a resting awake EEG recorded during the first months following a brain injury provides potentially useful information on the patient’s clinical outcome with regards to survival six months later.

## Conclusions

Taken as a whole, the present results suggest that resting awake EEG recorded during the first month following a brain injury can provide potentially prognostic valuable information on the patient’s outcome (survival or death) six months later, at least at the group level. Specifically (a) the diversity and the variability of EEG (measured by SPD and SPV), and (b) the probability of the occurrence of delta, slow- and fast-theta and alpha oscillations can be considered potential candidates for prognostic EEG markers that may allow us to predict the chances of survival in VS or MCS at group level.

To our knowledge this is the first study that demonstrates that a resting awake EEG may be useful in providing potentially prognostic information on the VS or MCS future outcome in terms of whether a patient will survive or die.

It is important to stress that many more studies are needed in order to provide more evidence. Future studies including a larger group of patients are required to confirm these results. If confirmed, then these potential candidates for prognostic EEG markers should be validated in the individual patients in VS or MCS.
